# Breast cancer screening knowledge among Hungarian women: a cross-sectional study

**DOI:** 10.1186/s12905-021-01204-9

**Published:** 2021-02-15

**Authors:** Diána Reményi Kissné, Noémi Gede, Zsolt Szakács, István Kiss

**Affiliations:** 1 Doctoral School of Health Sciences, University of Pécs, Pecs, Hungary; 2 Institute for Translational Medicine, Medical School, University of Pécs, Szigeti út 12., 7624 Pecs, Hungary; 3 János Szentágothai Research Center, University of Pécs, Pecs, Hungary; 4 Department of Public Health Medicine, Medical School, University of Pécs, Pecs, Hungary

**Keywords:** Breast cancer screening, Knowledge, Mammography

## Abstract

**Background:**

Breast cancer (BC) is the leading malignant tumor among women worldwide. Although attending regular BC screening effectively reduces cancer-related mortality, surveys testify that screening knowledge is critically low among women. We aimed to conduct a comparative cross-sectional survey to assess BC and BC screening-related knowledge in Hungary.

**Methods:**

Women between 25 and 65 years of age without a previous history of malignant tumors were included with non-probability sampling in 2017. Respondents were recruited either from primary care (laywomen) or from the waiting rooms of mammography (screening attendees). A self-completion questionnaire was constructed with questions about BC (risk factors, signs and symptoms, curability, and mortality), BC screening (mammography and breast self-examination), and BC-related information sources to assess knowledge among laywomen and screening attendees. In addition to descriptive statistics, odds ratios with 95% confidence intervals were calculated in univariate analysis and logistic regression was used in multivariate analysis.

**Results:**

Altogether, 480 women completed the questionnaire, of which 429 (227 laywomen and 202 screening attendees) were eligible for inclusion. Laywomen and screening attendees knew the recommended age at first mammography in 35.2% and 86.6%, the recommended frequency of screening in 33.9% and 12.9%, the recommended age at first breast-self examination in 38.8% and 51.2%, had sufficient knowledge of the risk factors of BC in 7.0% and 5.9%, and that of signs and symptoms of BC in 16.7% and 28.9%, respectively. A higher proportion of screening attendees correctly identified the recommended age of first BC screening correctly than that of laywomen (86.6% vs. 35.2%; *p* < 0.001). The most popular information sources were television among laywomen and general practitioners or specialists among screening attendees. In multivariate analysis, older age, higher education, and place of residency were significant predictors of the right answers.

**Conclusions:**

Although knowledge was insufficient in almost all fields of the questionnaire, the most prominent gap was observed concerning risk factors and signs and symptoms of BC both in laywomen and, unexpectedly, screening attendees. Most laywomen were lacking knowledge of screening protocol. These results urge breast health and BC knowledge interventions in Hungary.

**Supplementary information:**

The online version contains supplementary material available at 10.1186/s12905-021-01204-9.

## Background

Breast cancer (BC) was a leading malignant tumor regarding incidence, prevalence, and mortality among women worldwide as well as in Europe in 2018. Worldwide incidence of female BC was 2 million while BC was responsible for more than 0.6 million fatalities in 2018. Within Europe, BC incidence was 522,513 with more than 137,707 BC-related fatalities in 2018 [1]. In Hungary, annual BC incidence has been exceeding 8000 cases since 2015 and reached 8215 with a mortality of 2212 in 2018 [2]. Data testified that Hungary is considerably behind the EU average concerning standardized mortality ratio of BC in 2018 [1].

BC is a multifactorial disease with complex pathomechanisms. Risk factors of BC include but are not limited to positive medical history for conditions or disorders in obstetrics, gynecology, reproduction, and endocrinology. Age is deemed to be an independent risk factor of BC. Besides, the cumulative number of periods seems to be important, which supports the central role of endogenous estrogen in the pathogenesis. Risk of BC doubles in cases where the first delivery is later than 30 years of age [3]. Additional modifiable risk factors involve the exposure to certain exogenous hormones (e.g., postmenopausal hormone therapy applied frequently to relieve the undesired changes of menopause) [4], smoking [5], high-fat diet [6], being overweight or obese, sedentary lifestyle, alcohol consumption (more than 1–2 drinks daily), high socioeconomic status, oral contraceptives containing estrogen, progestagen, or their derivatives; and vitamin D deficiency [4]. Chest irradiation carries a delayed, 7- to 17-fold risk of BC [7–9]. Protective factors include suckling: 12-month breastfeeding reduces the risk of pre-menopausal BC by 4% [10]. Genetics may account for 5–10% of BC cases, of which even 30% may have BRCA 1 or BRCA 2 mutations [11]. These mutations increase the risk of BC tenfold, although several other genes (the so-called ‛BC genes’) have been implicated as contributors to the pathogenesis [12] Hereditary syndromes, e.g., Li-Fraumeni [13] and Cowden syndromes carry a high risk of BC [14].

A potential approach to coping with high BC-related mortality is the introduction of BC screening programs. Studies proved that BC public health interventions promote early detection. Screening uptake of at least 70% of the target population reduces BC-related mortality significantly [15]. Although mammography reduces BC-related mortality by a remarkable 25% among women between 50 and 69 years of age, the risk reduction is less prominent in younger women between 40 and 49 years of age [16]. On the contrary, studies recorded a humble 15% decrease in BC-related mortality with frequent overdiagnosis (30%) and, consequently, frequent overtreatment [17]. The success of BC screening can be attributed to early detection because the immediate removal of tumors with a diameter < 10 mm results in an average survival of 20 years in approximately 90% of the cases [18].

Mass screening for BC had been incorporated into public health services in Hungary in 2001 [15]. The target population of BC screening involves asymptomatic women between 45 and 65 years of age who are regularly invited to participate in screening with X-ray mammography once every two years [19, 20]. Hungarian data from 2015 revealed that invitation for BC screening reaches 78.5% of the target population. Digital mammography accounted for 60% of cases. These quality indicators are far behind the 2015 EU average [20].

There was not a comprehensive report which assessed women’s knowledge of the field in Hungary, while insufficient knowledge may contribute to the suboptimal screening attendance rate. This inspired us to assess the knowledge of BC and BC screening among Hungarian women. In addition, we aimed to explore which channels are used by women to gather relevant information.

## Methods

The study is reported following the STROBE Statement.

### Study design and settings

This study is a cross-sectional survey. Subjects were recruited from 12 general practitioner clinics and the outpatient Department of Radiology, University of Pécs, Medical School, Hungary. Recruitment period lasted from March 2017 to June 2017.

### Sample

We recruited a total of 480 women aged between 25 and 65 years with non-probability sampling (this age interval was the inclusion criteria). Missing data resulted in the exclusion of 52 women. Finally, data of 428 women (89.1%) with complete dataset were eligible for the analysis. The study population was divided into two groups by site of recruitment: 227 women were recruited from primary care (laywomen) and another 201 from the waiting rooms of mammography of the Department of Radiology (screening attendees). The exculsion critera was the history of malignant tumors.

### Questionnaire development and validation

We constructed a self-completion questionnaire. As the first step of production, our team of epidemiologists indicated the main areas of interest and phrased index questions accordingly. Then, the set of questions was revised by an oncologist to validate the medical content. Laypeople appraised the wording of questions. The English-language version of the questionnaire is provided in Appendix as Additional file.

The first set included 7 questions concerning sociodemography (sex, age, place of residence, marital status, education, financial situation, and religion). Followed by a set of 16 questions regarding knowledge of BC and BC screening, including multiple-choice questions about information sources. There were 2 multiple and 14 single choice questions given. In terms of signs and symptoms of BC, respondents indicating correctly at least 5 options of the 8 given (all options were true) were considered to have sufficient knowledge. In terms of risk factors of BC, respondents indicating correctly at least 2 options and incorrectly maximum 1 option of 21 given (13 and 12 were true in the groups of screening attendees and lay women, respectively) were considered to have sufficient knowledge.

Medical assistants distributed the questionnaire and obtained signed informed consent from participants. We secured anonymity of participants by linking informed consents to the corresponding questionnaires with a numeric code. Documents were kept in locked cabinets separately until processing.

### Statistical analysis

We performed descriptive statistics including the calculation of central tendencies (means or medians) with the measure of dispersion and relative frequencies. In univariate analysis, we used the Mann–Whitney U-test to examine the association between the level of education and knowledge of the risk factors of BC. We used the χ^2^ test with Z test to reveal the association between participation in screening and knowledge of timing of BC screening. In multivariate analysis, we used logistic regression with a probability of 95% with explanatory variables including age, education, and place of residency to examine the association between the sociodemographic characteristics and the dichotomous outcomes. Data were analyzed using SPSS 20.0 statistical software.

## Results

Baseline sociodemographic characteristics of the study population are summarized in Table 1. Main findings are summarized in Table 2.

**Table 1:** Sociodemographic characteristics of the groups

Variable	Laywomen	Screening attendees
	(N^0^ = 227)	(N^0^ = 202)
Age (mean)	47.22	53.33
(median)	48.00	55.00
(minimum)	25.00	31.00
(maximum)	64.00	64.00
Place of residence (N^0^, %)		
City town	117 (51.5%)	58 (28.9%)
Town	56 (24.7%)	74 (36.8%)
Village	54 (23.8%)	69 (34.3%)
Marital status (N^0^, %)		
Unmarried	23 (10.1%)	13 (6.5%)
Married/common-law marriage	136 (59.9%)	145 (72.1%)
Divorced/separated	47 (20.7%)	23 (11.4%)
Widowed	21 (9.3%)	20 (10%)
The highest level of education (N^0^, %)		
Less than primary school	2 (0.9%)	1 (0.5%)
Primary school	24 (10.6%)	18 (9%)
Vocational or industrial school	53 (23.3%)	43 (21.4%)
Secondary school	50 (22.0%)	81 (40.3%)
College or university	98 (43.2%)	58 (28.9%)
Health education (N^0^, %)		
Yes	140 (61.7%)	148 (73.6%)
No	87 (38.3%)	53 (26.4%)
Religiosity (N^0^, %)		
Yes	102 (44.9%)	113 (56.2%)
No	125 (55.1%)	88 (43.8%)
Employment status (N^0^, %)		
Employed	145 (63.9%)	119 (59.2%)
Unemployed	7 (3.1%)	4 (2.0%)
Inactive	66 (29.0%)	73 (36.3%)
Dependant	9 (4.0%)	5 (2.5%)
Financial situation (N^0^, %)		
Very bad	7 (3.1%)	1 (0.5%)
Poor	27 (11.9%)	15 (7.5%)
Average	108 (47.6%)	107 (53.2%)
Good	76 (33.5%)	74 (36.8%)
Very good	9 (3.9%)	4 (2.0%)

**Table 2:** Significant associated factors of respondents’ knowledge of BC screening, BSE and BC

Outcome	Laywomen		Screening attendees	
	Predictive factors	Effect	Predictive factors	Effect
Knowing the recommended age of first BC screening and the frequency of BC screening	Age	(ß = 0.039;* p* = 0.026; OR = 1.040 95% CI 1.005–1.076)	NS	
Knowing the recommended age of first BSE and the frequency of BSE	Education	(ß = 0.386;* p* = 0.016; OR = 1.472 95% CI 1.076–2.013)	NS	
Knowing that BC is a common cause of death in Hungary	Age	(ß = 0.029;* p* = 0.034; OR = 1.030 95% CI 1.090–1.058)	NS	
	Education	(ß = 0.279;* p* = 0.014; OR = 1.322 95% CI 1.059–1.651)	NS	
Knowing that there is an asymptomatic period of early BC	Age	(ß = 0.033;* p* = 0.043; OR = 1.033 95% CI 1.001–1.067)	Age	(ß = 0.046;* p* = 0.032; OR = 1.047 95% CI 1.004–1.093)
	Education	(ß = 1.057;* p* = 0.028; OR = 1.985 95% CI 1.090–3.615)	Education	(ß = 0.395;* p* = 0.015; OR = 1.485 95% CI 1.080–2.041)
	Place of residency (county town vs. village)	(ß = 0.577;* p* < 0.001; OR = 1.780 95% CI 1.342–2.360)	NS	
Being well-informed about symptoms	Education	(ß = 0.493;* p* = 0.001; OR = 1.638 95% CI 1.213–2.211)	Education	(ß = 0.353;* p* = 0.012; OR = 1.424 95% CI 1.081–1.876)

All outcomes are adjusted for age, education and residency

### Timing of BC screening

A higher proportion of screening attendees correctly identified the recommended age of first BC screening correctly (that is, 45 years of age in Hungary) than that of laywomen (35.2% vs. 86.6%; *p* < 0.001).

The recommended frequency of BC screening in average-risk women (that is, once every 2 years in Hungary) was identified correctly in 33.9% and 12.9% by laywomen and screening attendees, respectively. Comparing screening attendees to laywomen of screening age not attending BC screening in the past 2 years did not reveal a significant difference between groups. Respondents who knew both the recommended age of first BC screening and the recommended frequency of BC screening accounted for 16.7% and 10.0% of laywomen and screening attendees, respectively. Associated factors of knowing the right answers are listed in Table 2.

### Breast self-examination (BSE)

**Fig. 1:**
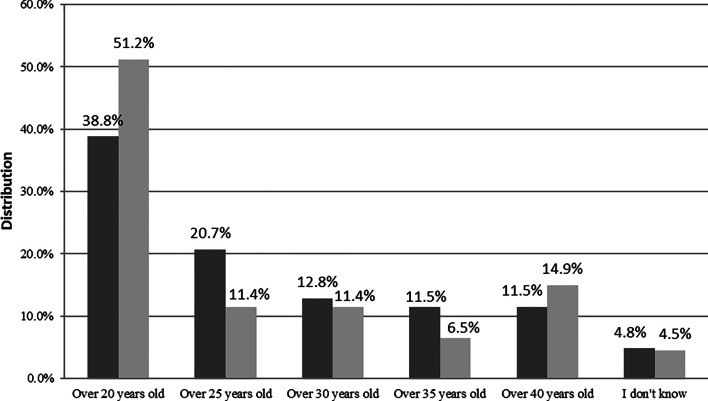
Age at first breast self-examination.  Laywomen,  Screening attendees

The recommended age of first BSE (that is, 20 years of age) was rightly recognized in 38.8% and 51.2% by laywomen and screening attendees, respectively (Fig. 1).

**Fig. 2:**
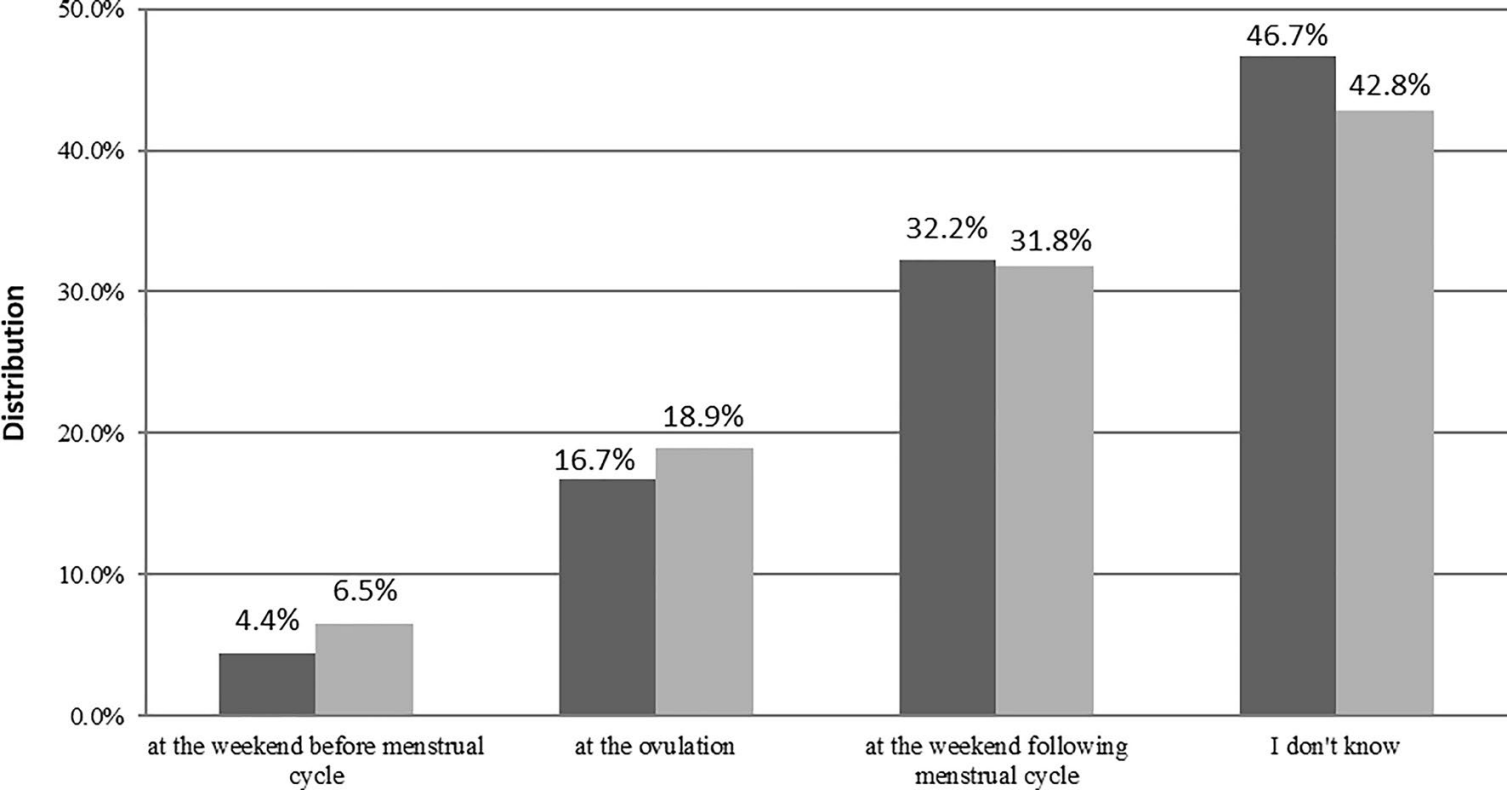
Timing of breast self-examination.  Laywomen,  Screening attendees

The recommended timing of BSE (that is, at the weekend following period) was identified correctly in 32.2% and 31.8% by laywomen and screening attendees, respectively; without a significant difference between screening attendees and laywomen of screening age without attending for BC screening in the past 2 years (Fig. 2). Respondents who knew both the recommended age of first BSE and the recommended timing of BSE accounted for 13.7% and 18.4% of laywomen and screening attendees, respectively. Associated factors of knowing the right answers are listed in Table 2.

### Curability and mortality of BC

The majority of laywomen believed that early BC can be curable (92.5%), the ratio was similar among screening attendees (96.5%).

Laywomen and screening attendees stated that BC is a common cause of death in Hungary in 68.8% and 76.6% respectively. Associated factors of knowing the right answers are listed in Table 2.

### Signs and symptoms of BC

An early BC can be asymptomatic according to 80.6% and 78.1% of laywomen and screening attendees, respectively.

**Fig. 3:**
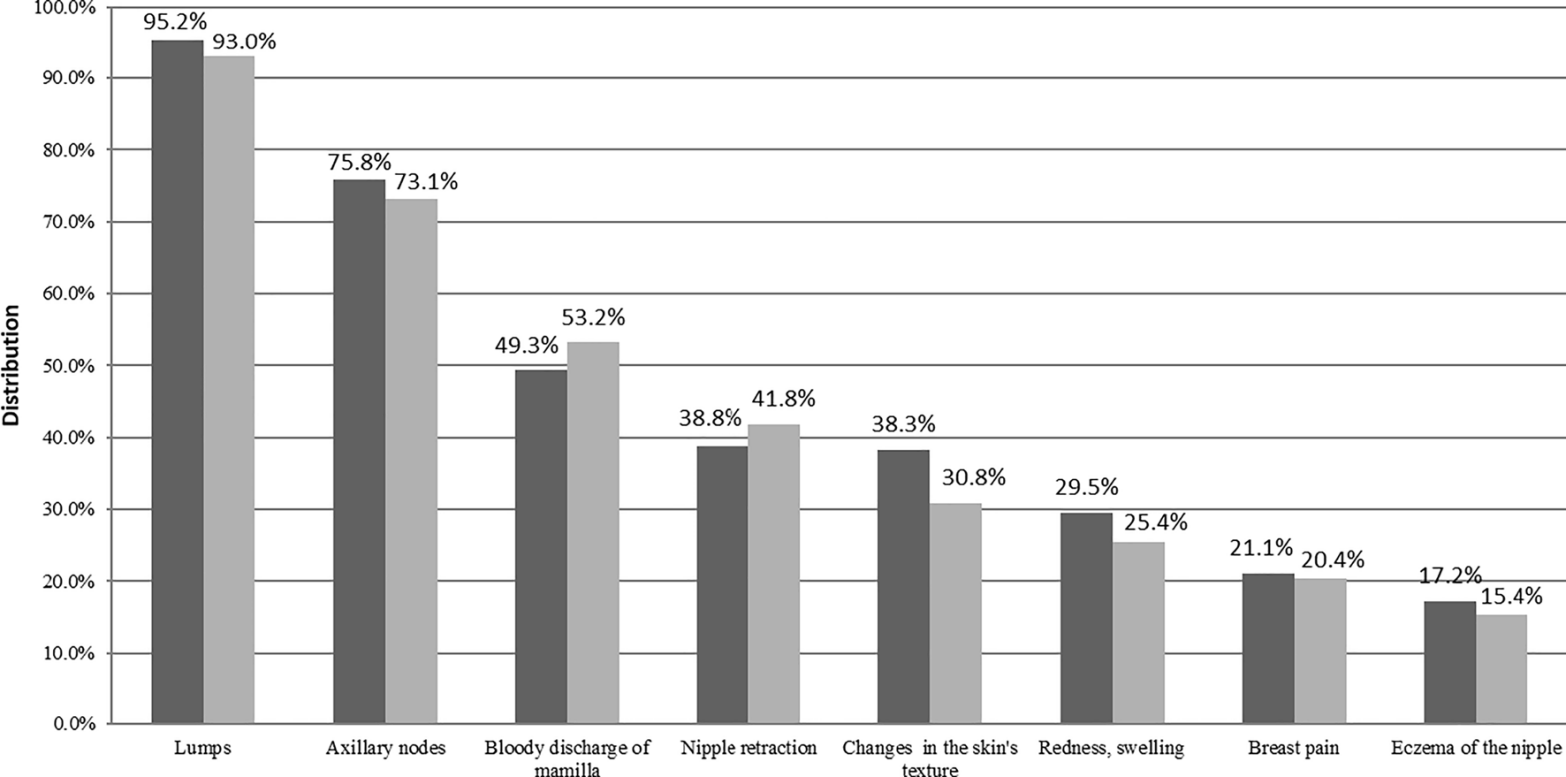
Signs and symptoms of breast cancer.  Laywomen,  Screening attendees

16.7% and 28.9% of laywomen and screening attendees had sufficient knowledge of symptoms, respectively. Agreement on the 3 most common symptoms was good between groups: lumps (95.2% and 93.0%), axillary nodes (75.8% and 73.1%), and bloody discharge of mamilla (49.3% and 53.2%) were indicated by laywomen and screening attendees, respectively (Fig. 3). Associated factors of having sufficient knowledge are listed in Table 2.

### Risk factors of BC

The majority of both laywomen and screening attendees had sufficient knowledge of risk factors of BC (7.0% vs. 6.0%, respectively). Laywomen believed that genetic predisposition (81.1%), physical trauma (55.5%), and smoking (47.1%) are the three most common risk factors, whereas screening attendees favored to choose genetic predisposition (85.6%), physical trauma (49.3%), and irradiation (45.8%). The data did not satisfy the conditions of logistic regression model because only a small proportion of the respondents had sufficient knowledge of risk factors so that we analysed this outcome with univariate statistics exclusively. Among screening attendees, women who had sufficient knowledge were better educated (*p* = 0.01).

### Source of information

**Fig. 4:**
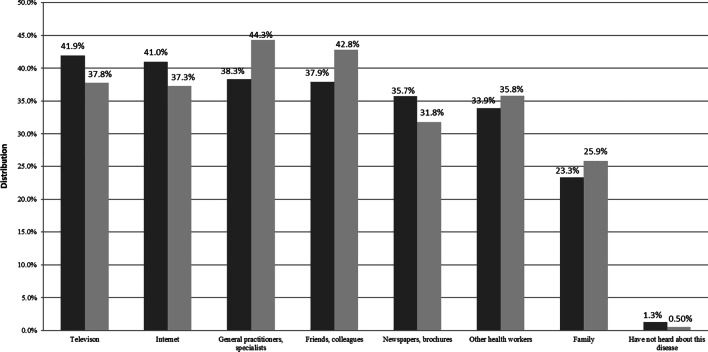
Information sources among laywomen and screening attendees.  Laywomen,  Screening attendees

Television (41.9%), the internet (41.0%), and general practitioners or specialists (38.3%) were the three most common information sources among laywomen. On the contrary, screening attendees favored to choose general practitioners or specialists (44.3%), friends and colleagues (42.8%), and television (37.8%) to be the three most common sources (Fig. 4).

## Discussion

This study aimed to assess the knowledge of women (laywomen vs. screening attendees) of BC and BC screening. Since screening uptake is suboptimal in Hungary, an improvement would be desirable to reduce BC-related mortality. One potential tool for increasing awareness would be the initiation of public health interventions.

As we expected, there was a prominent difference between groups in knowing the recommended age at first BC screening: the ratio of correct answers favored screening attendees over laywomen of screening age not attending for BC screening in the past 2 years (86.6% vs. 35.2%, respectively). This might be attributed to the invitation letters for BC screening when reaching the appropriate age of screening (i.e., 45 years in Hungary) [15]. The difference between groups highlights the efficacy of population-based invitation letters for mass screening, even if the entire target population is not covered (around 80%). However, we failed to detect a similar difference regarding the recommended frequency of BC screening; here, the ratio of correct answers was extremely low in both groups. The importance of screening is highlighted by the fact that the 5-year relative survival rate of stage III BC is about 72%, women in this stage can often be treated successfully. On the contrary, stage IV (metastatic) BC has a 5-year relative survival rate of about 22% [21]. Knowledge of BC screening protocol, particularly for those reaching the recommended age at first screening, should be improved. Among laywomen, respondents who had sufficient knowledge were older, which highlights the importance of education of the younger generation. The required information may be transmitted via the internet and the television for the younger women, and by family doctors and specialists or by distribution of flyers for the older women because our findings highlighted these as the most frequently chosen information sources.

Although BSE should be started more than two decades earlier than mammography (20 years vs. 45 years, respectively), most respondents were unaware of this. These results corroborate the findings of the study of Do Thi Thanh Toan et al. from North-Vietnam, in which only 19.3% of the respondents knew when to perform the first BSE [22]. Knowledge of screening attendees was not better significantly compared to that of laywomen of screening age not attending for BC screening in the past two years; this is true concerning the timing of regular BSE (i.e., at the weekend following period) as well. Although physical examination has a low sensitivity (54%), its specificity is high (94%) [23] and seems more relevant for women aged 40–49 years than those aged above 50 years [24]. Laywomen with better education were more likely to know the recommended age of first BSE and the recommended timing of BSE. Since our findings were disappointing regarding BSE, it would be important to include the most important features of this screening modality (i.e., technique, timing in the period, and from what age) in the sexual education program of primary schools. This preventive activity may facilitate the early recognition of BC.

More than 90% of respondents had sufficient knowledge of the curability of early BC. In a study from Beirut, respondents had severe knowledge gaps regarding curability but were well informed about signs and symptoms [25]. A Mongolian study resulted in results comparable to ours: 91.1% of respondents knew that early recognition of BC could improve survival [26]. On the contrary, only about 70% of the respondents knew that BC is a common cause of death. In Hungary, BC is the most common malignant tumor and the third most common cause of tumor-related mortality among women [1, 2]. Higher education and older age seem to be associated with better knowledge in laywomen.

About 80% of respondents knew that BC can be asymptomatic. However, both laywomen and screening attendees had insufficient knowledge of the typical clinical presentation: less than one-fourth of the respondents proved to have sufficient knowledge of signs and symptoms of BC. It would be important to extend women’s knowledge of this aspect because recognizing signs and symptoms is a key moment in the detection and effective therapy of early BC. Literature states that the common signs and symptoms are lumps, mastitis, breast pain, and mamillar discharge [27]. The answers of respondents from the groups partly overlap: lumps were indicated by almost all respondents, whereas only half of them indicated mamillar discharge. Moreover, more than 70% indicated that axillary nodes belong to common symptoms while lymphatic metastases are rather characteristic of advanced BC. It is important to highlight that the third most frequently indicated symptom was the bloody nipple discharge, indicated approximately by half of the respondents in both groups. In the study of Linsell et al. from the UK, most women (85%) knew that lumps and axillary nodes could be the signs of BC, but less than half of respondents knew that BC could be associated with non-nodular signs as well [28]. The attendance rate of mammography can be increased only if women are aware of the warning signs and symptoms of BC; otherwise, they remain unrecognized and women will not see the doctor. Again, higher education, older age, living in county town were amenities, as demonstrated concerning signs and symptoms. According to a report, a positive family history for BC is associated with better knowledge (Western Turkey; 2011) [29]. According to our survey, BC among friends proved to be an associated factor of correctly knowing the frequency of BC.

A dramatically insufficient knowledge was explored regarding the risk factors of BC: less than 10% of respondents had sufficient knowledge. Opposing to the low knowledge in the Hungarian cohort of subjects, Trupe et al. demonstrated that about one-third (31.3%) of respondents had sufficient knowledge of risk factors of BC (in South African women aged 18–68 years; 2017) [30]. In our survey, both laywomen and screening attendees chose the same answers to be the two most common risk factors: genetic predisposition (81.1% vs. 85.6%, respectively) and physical trauma (55.5% vs. 49.3%, respectively). Discrepant answers were given regarding the third most common factor: laywomen favored smoking (47.1%), whereas screening attendees favored irradiation (47.1%). In an Indian study, respondents listed smoking and alcohol consumption as risk factors of BC, but many respondents highlighted the importance of positive family history for BC [31]. The role of genetics and irradiation was overestimated by the respondents: in fact, genetic predisposition is responsible only for around 5–10% of all BC cases, the most common association is with the mutations of BRCA genes [11]. Most respondents were misinformed about the role of physical trauma: it has not been proven without doubt that trauma increases the risk of BC [32]. Screening attendees who had sufficient knowledge were significantly better educated.

The most popular information sources were television and the internet among laywomen, whereas screening attendees favored general practitioners or other specialists, and colleagues or friends. Among adult Nigerian women (2014), the most common sources of information were the media and health care workers [33]. According to the survey of Maloney EK et al., laywomen preferred the internet to doctors to gather information about BC screening. Respondents frequently searched for information from the websites of cancer organizations about alternative therapies, adverse effects of therapies, conventional therapies, and traditional therapies (in Americans aged 27–79 years; 2015) [34]. Although the reliability and credibility of websites are questionable and often misleading, it is rare that patients, or at least their relatives, do not search for information about BC on the internet. The discrepant information sources between groups may explain the differences in the answers to the questions related to screening protocol and clinical phenotype of BC.

### Limitations

Cross-sectional surveys do not permit causal generalizations. Another limitation of the study is the nonprobability sampling used, raising concerns about self-selection bias.

## Conclusions

Our results revealed that Hungarian women including laywomen and, unexpectedly, screening attendees are often mis- and underinformed about the risk factors as well as about the signs and symptoms of BC. Most laywomen are lacking knowledge of screening protocol. These findings urge for immediate BC screening and breast health knowledge intervention to increase knowledge among people, especially in the younger and less educated strata of society and villagers. Since electronic media (among laywomen) and healthcare workers (among screening attendees) are the major information sources, distribution of reliable and easily digestible information via these channels may improve knowledge, therefore improving awareness of BC screening. Our findings implicate that additional education may be recommended for BC screening attendees.

## Supplementary Information

**Additional file 1:** Questionnaire.

## Data Availability

The datasets used and/or analysed during the current study are available from the corresponding author on request.

## Abbreviations

BC: Breast cancer
OR: Odds ratios
CI: Confidence intervals
BSE: Breast self-examination
