# Supplementary material for: Breast cancer screening knowledge among Hungarian women: a cross-sectional study
Source: BMC Womens Health. 2021 Feb 15;21:69. doi: 10.1186/s12905-021-01204-9 (PMC7885515; doi:10.1186/s12905-021-01204-9)
Supplement: Supplementary file 1 — Additional file 1: Questionnaire. [file 12905_2021_1204_MOESM1_ESM.docx]

1. **Age:** ………… years
2. **Where do you live?**

O City town

O Town

O Village

1. **What is your marital status?**

O Unmarried

O Married/Common-law marriage

O Divorced/Separated

O Widowed

1. **What is your highest level of education?**

O Not completed primary school

O Primary school

O Vocational school / Industrial school

O Secondary school

O College/University

1. **What is your employment status?**

O Employed

O Unemployed

O Inactive

O Dependent

1. **How do you judge your financial situation?**

1. **Are you religious?**

O Yes

O No

1. **What is the recommended age of first attendance for breast cancer screening in an average-risk population?**

O above the age of 25 years

O above the age of 35 years

O above the age of 45 years

O above the age of 55 years

O above the age of 65 years

O I do not know.

1. **How often is the participation in breast cancer screening recommended for the average-risk population?**

O Every half a year

O Annually

O Biannually

O Every three years

O I do not know.

1. **What is the recommended age of the first breast self-exam in an average-risk population?**

O above the age of 20 years

O above the age of 25 years

O above the age of 30 years

O above the age of 35 years

O above the age of 40 years

O I do not know.

1. **What is the appropriate timing of a breast self-exam?**

O Every month, before period on the weekend

O Every month, mid-cycle

O Every month, after period on the weekend

O I do not know.

1. **Is early-stage breast cancer curable?**

O Yes

O No

O I do not know.

1. **Do you have friends who suffered from breast cancer?**

O Yes

O No

O I do not know.

1. **Can early-stage breast cancer be asymptomatic?**

O Yes

O No

O I do not know.

1. **From which sources do you gather information about breast cancer? (You can indicate multiple answers)**

☐ General practitioners, specialists

☐ Other health workers (nurse, assistants, etc.)

☐ Friends, colleagues

☐ Family members

☐ Television

☐ Internet

☐ Newspapers, brochures

☐ Other:…………………………..

☐ I have not heard about breast cancer at all.

1. **Please, choose risk factors contributing to the development of colorectal cancer. (You can indicate multiple answers)**

☐ Low intake of fruit and vegetables, and high-calorie diet, particularly fat-rich

☐ Alcohol abuse

☐ Traumatic breast injury

☐ Age above 55 years

☐ Late menopause

☐ Being overweight

☐ Smoking

☐ High blood pressure

☐ Hormone therapy after menopause

☐ Sedentary lifestyle

☐ Oral contraceptives (‘pills’)

☐ Use of painkillers

☐ Radiation to the chest

☐ Early period

☐ Hereditary predisposition

☐ High cholesterol level

☐ HPV-infection

☐ Diabetes

☐ Strong ultraviolet radiation

☐ Breastfeeding

☐ Childlessness

1. **Please, choose the most typical symptoms of colorectal cancer. (You can indicate multiple answers)**

☐ Lumps

☐ Breast or nipple pain

☐ Nipple retraction or inversion

☐ Dimpling

☐ Nipple discharge

☐ Swelling

☐ Redness

☐ Changes to the skin’s texture

1. **Is breast cancer a frequent cause of cancer death in Hungary?**

O Yes

O No

O I do not know.
